# Does Kirchhoff’s Law Work in Molecular-Scale Structures?

**DOI:** 10.1021/acsomega.4c09854

**Published:** 2025-02-27

**Authors:** Abdullah Alshehab, Ali K. Ismael

**Affiliations:** † Physics Department, College of Science, King Faisal University, Al Ahsa 31982, Saudi Arabia; ‡ Department of Physics, Lancaster University, Lancaster LA1 4YB, U.K.

## Abstract

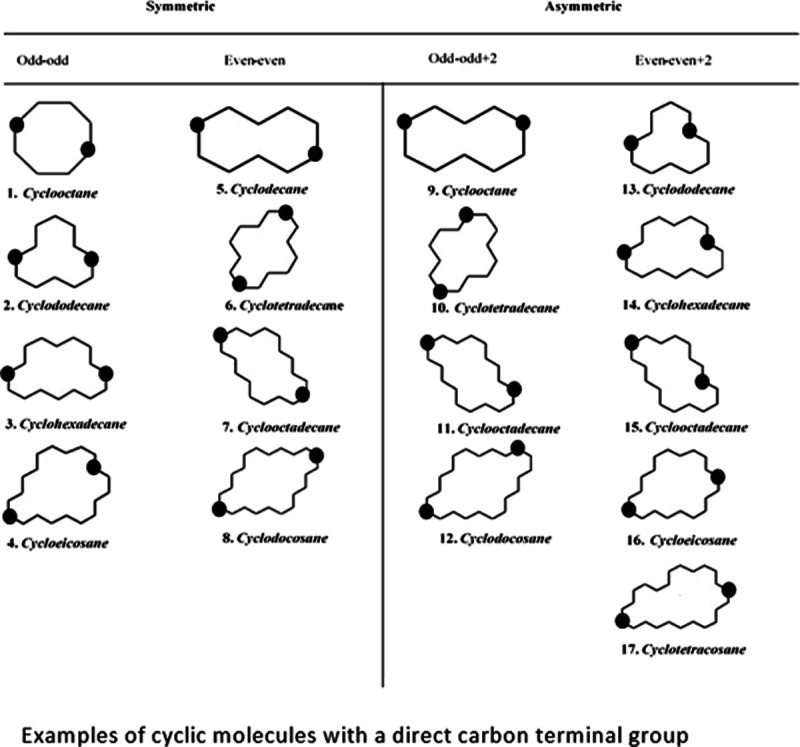

This study aims to theoretically and comprehensively investigate the single-molecule electrical conductance of symmetric and asymmetric alkane cyclic (*SAC* and *AAC*) molecules and their corresponding linear chains with three different terminal end groups including thiol (−*SH*), direct carbon (−*C*), and amine (−*NH*_2_). Here, we examine the validity of Kirchhoff’s law concerning sigma nonconjugated molecules at the nanoscale level. Counterintuitively, the electrical conductance (*G*) of symmetric and asymmetric alkane cyclic molecules with two parallel conductance paths is lower than that of their corresponding single chains with only one conductance path. This completely contradicts classical rules for combining conductances in parallel, regardless of the anchor group type, in light of this study’s use of symmetric and asymmetric cyclic molecules. A comparison of the DFT prediction trends with scanning tunneling microscopy measurements indicates that they are well-supported. The results of this investigation demonstrate an excellent correlation between our simulations and experimental measurements, for both SAC and AAC structures of different cavity size *n*,*m* = 3,3; 4,4; 5,5···10,10 and *n*,*m* = 3,5; 4,6; 5,7; 6,8; 7,9; 8,10; and 9,11 and for three different terminal end groups.

## 1 Introduction

The transport of electrons through molecular junctions has been extensively investigated, experimentally and theoretically. Such a transport system is critical to electronic development in the future, and it could have several applications.^1^ Many organic and inorganic molecules have been studied intensively over the past few decades.^2−8^ Several significant phenomena have been observed in molecular electronics, including switching, rectifying, and negative differential resistance. In light of these findings, it is clear that this area has significant potential and a promising future.^9−17^ Molecular devices are believed to replace traditional electronic components in the construction of molecular computers, which is a dream of many scientists. However, it should be noted that molecular device research is still at a young stage and is subject to a number of experimental and theoretical challenges.^1^ Besides experimental studies, theoretical calculations of molecular devices based on well-defined structural properties of nanoscale molecules contribute to understanding molecular conductance.^18−23^

Classically, based on Kirchhoff’s law of superposition, using two identical conductors in parallel produces a total conductance that is twice that of a single conductor. On the other hand, in the nanoscale world, parallel connections of two conductors within a molecular circuit rarely produce a conductance governed by such a classical superposition law. This is because electron tunneling through a molecular circuit obeys quantum physics laws rather than classical statistics physics laws. The total conductance was reported in an early theoretical study^24^ as the sum of the conductances of the individual branches (*G*_1_ and *G*_2_), coupled with an interference term
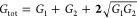
1

In the case of identical conductors, the conductance should be multiplied by a factor of 4 rather than two, as in classical conductance calculations. Vazquez et al.^25^ have accomplished experimental validation of this prediction by measuring the conductances of four pairs of single-backbone and double-backbone molecular junctions and determining that their conductance ratios ranged from 2.8 to 1.6. Thus, the ratios ranged from greater than to less than the classical value of 2, but no ratio exceeded four.

Remarkably, electron transport through the sigma systems with nonconjugated molecules has been neglected. The experimental measurements were concentrated on quantum interference within the pi system of multibranch and conjugated molecules; recent developments in molecular-scale transistors highlight the importance of sigma-mediated transport, in which a self-assembled monolayer (SAM) is found to have a 100 times higher on/off ratio than a SAM formed of conjugated molecules at room temperature.^17,25,26^ Consequently, sigma systems of multibranch molecules have attracted a growing amount of attention in recent years. For instance, an early theoretical and experimental study suggested that the electrical conductance of alkane rings decreases with increasing ring size.^27−30^ Charge transport via sigma systems with alkane rings has recently been studied theoretically and experimentally. A study^31^ investigated the conductance of heterocyclic alkanes compared to their linear alkane chain counterparts. As a result of the scanning tunneling microscopy-based break junction (STM-BJ) method and calculations based on density functional theory (DFT), it was reported that three heterocyclic alkanes, piperazine, C_222_-diaza, and dithiane, had conductance values that were lower than those of analogous linear alkane chains. Hence, organic molecules can design short molecular insulators through heterocyclic alkanes with destructive quantum interference. The conductance of sigma systems tends to be low, for instance, in heterocyclic alkanes, due to the absence of the pi channel and the suppression of the transmission through the sigma channel.

It has been shown in a number of theoretical and experimental studies that an increase in the molecular length has a significant impact on the conductance of molecular junctions. According to the results of a theoretical investigation,^1^ which utilized DFT in combination with Green’s function and supported some experimental results, a number of molecular junctions were investigated for their conductance, namely, alkanedithiols, oligo (1,4 phenylene-ethynylene), and 1,4 benzenes (*n*-alkylthiol) (BDnT). It has been observed that alkanedithiol molecular junction conductance values decrease with an increase in the molecule length. Experimental data^32−37^ is in excellent agreement with these values. Conductance values of the OPEs, having one to five phenyl rings, decrease with increasing length, just as in alkanedithiols. There is good support for this theoretical trend in the experimental results. The OPE-1 molecule, for example, has an experimental conductance of 2.5 × 10^–4^*G*_o_,^38^ while OPE-2, with a length of 20 Å, has a conductance of 1.68 × 10^–4^*G*_o_.^39^ BDnT, which occurs when a phenyl ring is inserted into an alkyldithiol wire, displays a previous theoretical trend. This means conductance values decrease with increasing molecule length. A study of the electrical conductance of cyclic molecules is important not only due to the fact that they possess multiple electron transport paths, leading to nonclassical behavior, but also because cyclic molecules of varying sizes have the capability to bind selectively to analyte molecules, which could be used for sensing applications.^27^

In this research, we try to clarify that the nature or type of anchor groups that bind molecules to metal electrodes also significantly influence the transport through single-molecule junctions.^40−43^ This study investigates anchoring groups for their effect on single-molecule conductance by using alkane cyclic molecules as a model structure. This work began by exploring the conductance (***G***) of symmetric and asymmetric cyclic molecules of alkanes in an attempt to understand the ***G*** of alkane molecules using three different anchor groups: amine (−*NH*_2_), thiol (−*SH*), and direct carbon contact (−*C*). This study also aims to validate Kirchhoff’s law on the nanoscale level using some cyclic molecules with three different terminal groups and compare our results against published experimental measurements.

## 2 Methods

Initially, we modeled terminal group–Au binding and then relaxed each molecule in the existence of two fixed leads. Using the density functional code SIESTA,^44−49^ we gained the optimum geometries of isolated cyclic molecules by relaxing them until all forces on the atoms were not more than 0.01 eV/Å and one *k*-point (see Tables S1–S6 of the Supporting Information). A 250 Ry energy cutoff defined the real space grid using a double-ζ plus polarization orbital basis set, norm-conserving pseudopotentials, and the local density approximation (LDA) exchange correlation functional. In addition to using GGA, we also computed results using LDA and found that the transmission functions were comparable to those obtained using LDA.^50,51^ For the purpose of simulating the likely contact configuration in a break-junction experiment, we utilized leads composed of six layers of Au with 30 gold atoms in each layer and a pyramid of gold atoms at the end. A quantum transport code known as Gollum^52^ was applied to calculate the electrical conductance after each molecular junction was relaxed (more details can be found in Sections S4 and S5 of Supporting Information).

The current research examines 51 molecules with three different anchor groups. Table 1 shows some cyclic molecules with a direct carbon terminal group. Odd–odd and even–even molecules represent symmetric cyclic molecules (first and second columns, respectively). Odd–odd + 2 and even–even + 2 molecules represent asymmetric cyclic molecules (third and fourth columns, respectively) (examples of cyclic molecules with thiol and amine terminal groups are shown in Section S1 and Tables S1–S6 of the Supporting Information). The three terminal end groups of all studied cyclic molecules are connected to gold electrodes through an amine anchor (Au–*NH*_2_), a thiol anchor (Au–*S*), and a covalent bond to form a direct contact with carbon (Au–*C*). Figure S2 shows examples of three different anchor groups for cyclic molecules.

**Table 1:**
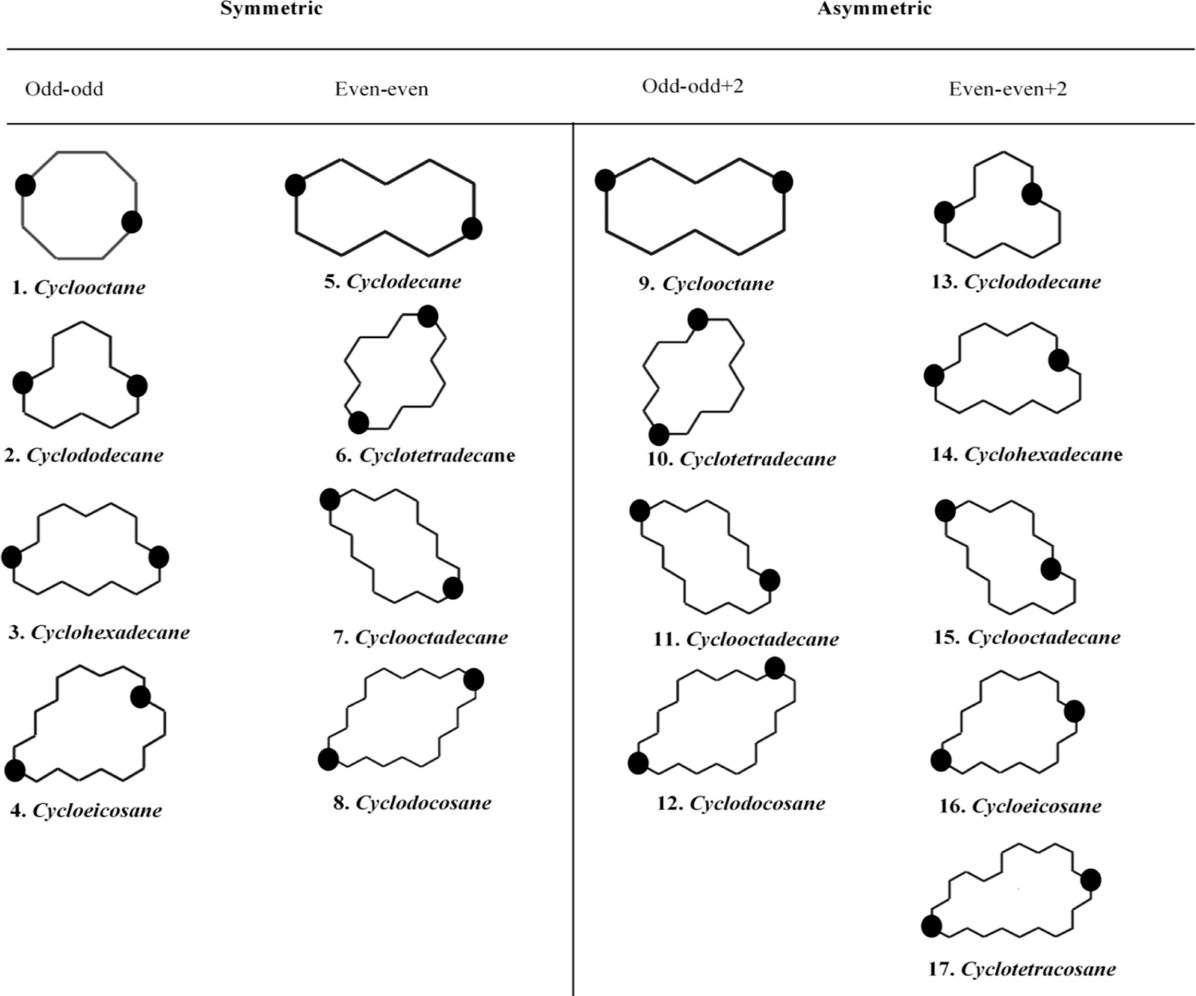
Examples of Cyclic Molecules with a Direct Carbon Terminal Group^a^

a Symmetric alkane cyclic molecules are represented by odd–odd and even–even molecules (first and second columns, respectively). Asymmetric alkane cyclic molecules are represented by odd–odd + 2 and even–even + 2 molecules (third and fourth columns, respectively). Note: The black solid circles represent the connectivity position of molecules to the metal electrodes at molecular junctions.

As a convenient way of discussing the conductance of double-branch cyclic molecules, we assign two integers, *n* and *m*, to represent the branch lengths of the number of methylene–*CH*_*2*_ units. Our approach involved forming a series of *C*_*n*_*C*_*m*_ rings with two branches *n,m* = *3,3; 3,5; 4,4; 4,6; 5,5; 5,7; 6,6; 6,8; 6,10; 7,7; 7,9; 8,8; 8,10; 9,9; 9,11; 10,10;* and *10,12* using three different anchor groups. For the sake of simplicity, we classified the molecules we studied into symmetric and asymmetric alkane cyclic molecules due to the fact that we have a large number of molecules. In *C*_*n*_*C*_*n*_ cyclic molecules, *–CH*_*2*_ units are only present in an even–even or odd–odd number. *C*_*n*_*C*_*m*_ asymmetric molecules, on the other hand, have even–even + 2 or odd–odd + 2 integers of *–CH*_*2*_ units (see Supporting Information, Tables S1–S6).

Continuing with the discussion, we examine 51 double-branch cyclic molecules. Different binding energies are necessary to bind the gold electrodes to these terminal groups. With a binding energy of −1.9 eV, the direct carbon anchor has the largest binding energy, followed by the thiol anchor at −1.7 eV and amine anchor at −0.3 eV, which is considered the lowest binding energy (see Supporting Information Figure S1).

## 3 Results and Discussion

Combining DFT with quantum transport theory has been used to model the transport properties of 51 alkane cyclic molecule junctions involving three different type of anchor groups. At the terminal of Au–*NH*_*2*_, the covalent bond distance is 2.8 Å. In the case of the *S* terminal, the Au–*S* distance is 2.3 Å and the Au–*C* distance is 2.3 Å for the terminal Au–*C* as illustrated in Figure S1 (more details can be found in Section S3 of the Supporting Information). The first step in this study is to examine transport across cyclic molecules at Au–Au junctions. In each anchor group, a unique type of transport is exhibited along with a particular trend in conductance.

The validity of Kirchhoff’s law can be demonstrated here using cyclic molecules with two parallel conductance paths at the nanoscale concerning sigma nonconjugated molecules with three different anchors. Table 2 shows a comparison of conductance between linear chains (*n*) and two parallel-path cyclic molecules (*n*,*m*), with thiol anchors (−*SH*). In the sigma system, Kirchhoff’s law is not applicable, which implies the conductance of linear chains (the third column from the left) is greater than the conductance of their corresponding rings (the eighth column from the left), for theoretical simulation. The same holds true for the STM measurements (see the fourth and last column). In addition, the conductance values calculated by Kirchhoff’s formula (fifth and sixth column from the left) do not correspond to the DFT values of full rings conductance nor do they correspond to the STM measurements (eighth and ninth columns, respectively).

**Table 2:** Conductances of Linear Chains (*G*_1_ and *G*_2_) and Symmetric Cyclic Molecules Were Determined from DFT Calculations and STM Experimental Measurements of the Thiol Anchor^a^

anchor	*n*	DFT log(*G*/*G*_o_)	STM log(*G*/*G*_o_)	DFT *G* = *G*_1_ + *G*_2_	STM *G* = *G*_1_ + *G*_2_	*n,m*	DFT log(*G*/*G*_o_)	STM log(*G*/*G*_o_)
–*SH*	3	–1.57		–1.23		3,3	–2.50	
	4	–2.00		–1.69		4,4	–2.69	
	5	–2.54		–2.25		5,5	–3.54	
	6	–3.00	–3.00	–2.69	–2.69	6,6	–4.00	–4.20
	7	–3.30		–3.00		7,7	–4.23	
	8	–3.80	–3.69	–3.52	–3.39	8,8	–4.12	–4.94
	9	–4.14		–3.85		9,9	–5.50	
	10	–4.65	–4.69	–4.4	–4.39	10,10	–5.47	–5.82

a The third and fifth columns represent the DFT calculations from ref (53), while the fourth and sixth columns represent experimental results from ref (27). Note: *n* for linear chains, while *n,m* for cyclic molecules.

In Table 2, it can be seen that Kirchhoff’s equation (fifth column) and full ring conductance (eighth column) are mathematically related
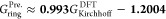
2where *G*_ring_^Pre.^ and *G*_Kirchhoff_^DFT^ are the conductance values resulting from applying Kirchhoff’s law and predicted conductance values of full rings (fifth and eighth columns), respectively, along with the thiol terminal group (−*SH*).

It is also possible to mathematically correlate Kirchhoff’s equation values and STM measurements by applying the following general formula
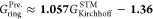
3where *G*_ring_^Pre.^ and *G*_Kirchhoff_^STM^ values represent, respectively, the ring total conductance calculated using Kirchhoff’s law with the thiol terminal group and the predicted conductance values with STM. To demonstrate the effectiveness of eq 3 by providing a quick test against published investigation,^20^ conductance of dithiane (C_2_C_2_) and its corresponding linear chain C_2_ was examined. Dithiane (C_2_C_2_) and C_2_ logarithmic conductance values were found to be −3.5 and −2.69. However, eq 3,predicts the *G* of dithiane to be −3.7. This demonstrates how powerful this equation is. As can be seen from eqs 2 and 3, the DFT calculations and the STM measurements agree well on the slopes. However, they have different *Y* intercepts. In Section S6 of the Supporting Information, we provide additional evidence to support the claim that Kirchhoff’s law is not applicable for cyclic molecules at the nanoscale level using another end group mainly direct carbon and amine anchors.

**Figure 1:**
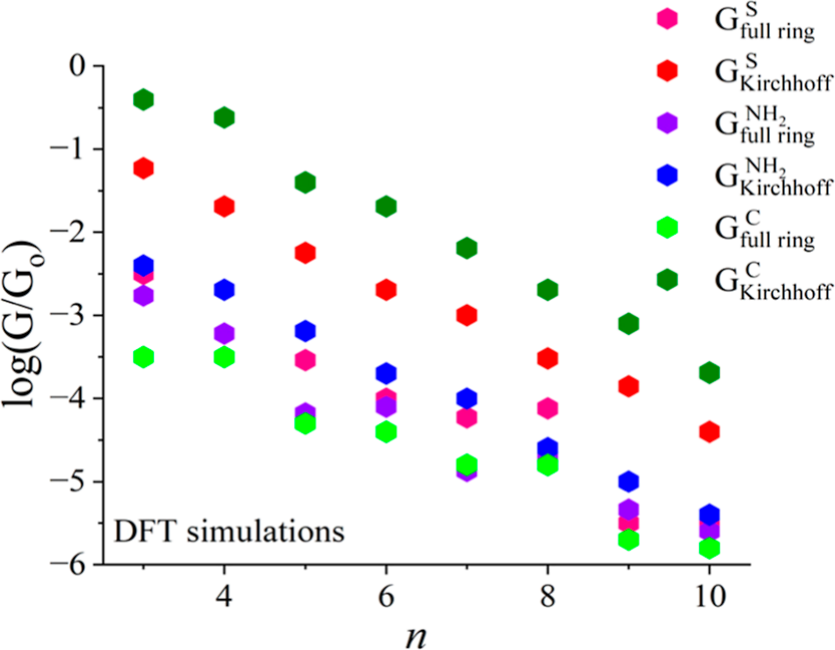
Validation of Kirchhoff’s law using 48 linear and symmetric cyclic molecules of three different terminal groups. A logarithmic conductance of full rings was compared with a logarithmic conductance derived by Kirchhoff’s law of three terminal groups: thiol, amine, and direct carbon contact. The dark green hexagons for direct carbon anchors represent Kirchhoff’s formula *G* values, while the light green hexagons for full rings *G* values. The red hexagons represent Kirchhoff’s values with thiol anchors, while the pink hexagons represent *G* values for full rings with the same anchor. In the same way, the blue and purple hexagons display Kirchhoff’s and full rings with amine anchors, respectively.

Figure 1 illustrates the validation of Kirchhoff’s law using symmetric cyclic molecules for three different terminal groups. Here, we compare a logarithmic conductance for full rings (***G***_**full ring**_), against the logarithmic conductance derived by Kirchhoff’s law (***G***_**Kirchhoff**_), and for thiol, amine, and direct carbon contact (***G***^***S***^**, *G***^***NH***_**2**_^**,** and ***G***^***SC***^). As an example, the red hexagons, representing G values for rings terminated with a thiol anchor, are higher than the pink hexagons, representing *G* values obtained by Kirchhoff’s formula. These patterns are also accurate when using the same rings with amine or direct carbon terminal groups. Through these thorough systematic simulations (48 molecules), we prove that the classical Kirchhoff’s law is invalid at the nanoscale for symmetric cyclic molecules. Kirchhoff’s law formula yields conductance values that are still higher than those of full rings. This nonclassical behavior is true with three different terminal groups as shown in Figure 1 below.

**Figure 2:**
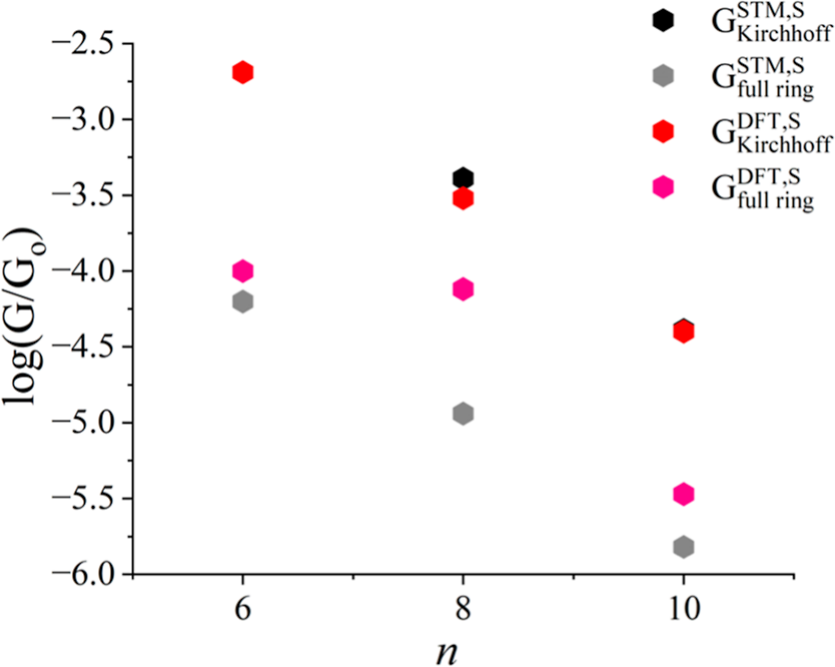
Validation of Kirchhoff’s law using symmetric cyclic molecules with a thiol anchor. A logarithmic conductance of full rings was compared with a logarithmic conductance derived by Kirchhoff’s law with thiol terminal groups theoretically and experimentally. The red hexagons represent DFT Kirchhoff’s formula *G* values, while pink hexagons represent DFT *G* values for full rings with a thiol anchor. The black hexagons indicate STM Kirchhoff’s formula *G* values, while the gray hexagons indicate STM *G* values for full rings with the same anchor. Note: The experimental measurements are described in ref (18).

A conductance comparison of our DFT predictions and STM measurements^18^ is shown in Figure 2 to support the theoretical DFT simulations. Figure 2 shows that DFT and STM conductance values coincide when Kirchhoff’s formula is used in the case of symmetric cyclic molecules with a thiol terminal group. As far as full rings are concerned, the DFT calculations are slightly higher than those obtained from STM measurements. We can observe excellently coincidental trends both theoretically and experimentally, which demonstrate the accuracy of our DFT results. In other words, the logarithmic conductance values of full rings are lower than the logarithmic conductance values derived by Kirchhoff’s law. As a result, Kirchhoff’s law does not apply in the nanoscale systems of symmetric cyclic molecules.

**Figure 3:**
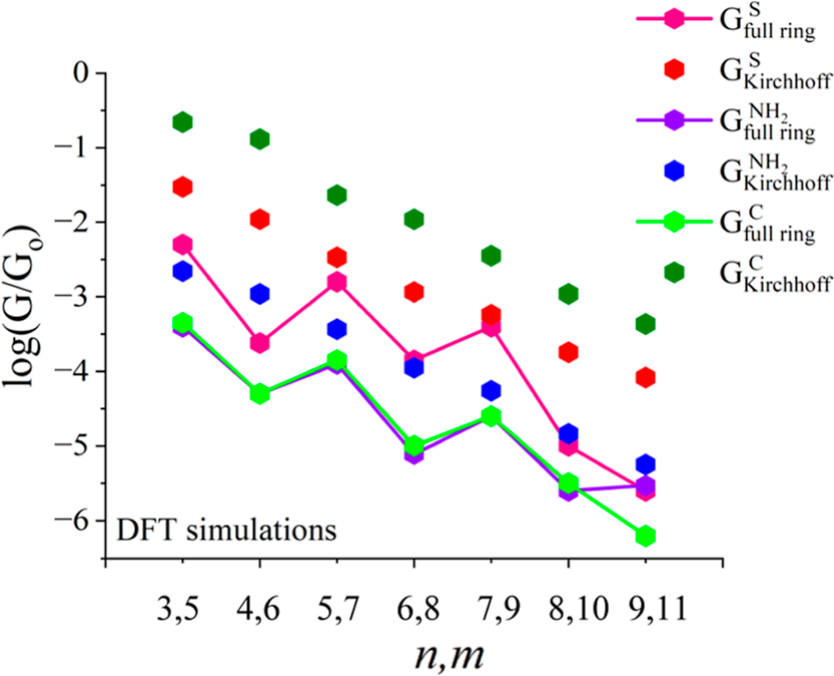
Validation of Kirchhoff’s law using asymmetric cyclic molecules for three different terminal groups. A logarithmic conductance of full rings was compared with a logarithmic conductance derived by Kirchhoff’s law with thiol, amine, and direct carbon contact. The dark green for direct carbon anchors represents Kirchhoff’s formula *G* values, while the light green line for whole rings represents DFT *G* values. The red hexagons represent Kirchhoff’s values with thiol anchors, while the pink line represents DFT *G* values for whole rings with the same anchor. In the same way, the purple-blue hexagons and purple line display Kirchhoff’s values and *G* values for full rings with amine anchors, respectively.

Investigating symmetric and asymmetric cyclic molecules alone with three different anchors can give an in-depth understanding of the conductance pattern. In Figure 3, Kirchhoff’s law for three terminal groups is validated using asymmetric cyclic molecules (*n*,*m*). Again, Kirchhoff’s law formula yields conductance values significantly higher than those of full rings with all terminal groups examined in this study (specifically, for the direct carbon contact). Our findings demonstrate that Kirchhoff’s law is invalid for both symmetric and asymmetric cyclic molecules at the molecular scale.

Figure 3 also shows that asymmetric cyclic molecules with thiol anchors exhibit a periodic conductance pattern with a sinusoidal function. The trend is nonconventional, which means, for example, C_5_C_7_ has a larger conductance (larger cavity) than C_4_C_6_ (smaller cavity). Conspicuously, odd-numbered asymmetric rings with the general form ***C***_2***n***–1_***C***_***m***=2***n***+1_ have a higher conductance than even-numbered asymmetric rings with the general form ***C***_2***n***_***C***_***m***=2***n***+2_. In other words, ***C***_2***n***–1_***C***_***m***=2***n***+1_ rings represent the crest (higher conductance) of the sinusoidal function, while ***C***_2***n***_***C***_***m***=2***n***+2_ rings represent its troughs (lower conductance), as shown in Figure 3. This periodic conductance pattern is still true with the same molecules with amine and direct anchor groups (−*NH*_2_ and −*C*). One possible explanation for the higher conductance of odd-numbered asymmetric rings than even-numbered asymmetric rings is the conformation of the molecules. The even-numbered asymmetric rings are less planar than the odd-numbered rings. Therefore, rings with a more planar conformation possess a higher conductance.^19^

The conductance of full asymmetric cycloalkane rings with thiol anchors is higher than that of full rings with amine and direct carbon anchors. For asymmetric cyclic molecules terminated with amine and direct carbon anchors, their conductances are approximately equal. The pattern is also correct for symmetric cyclic molecules, as shown in Figures 3 and 1.

**Figure 4:**
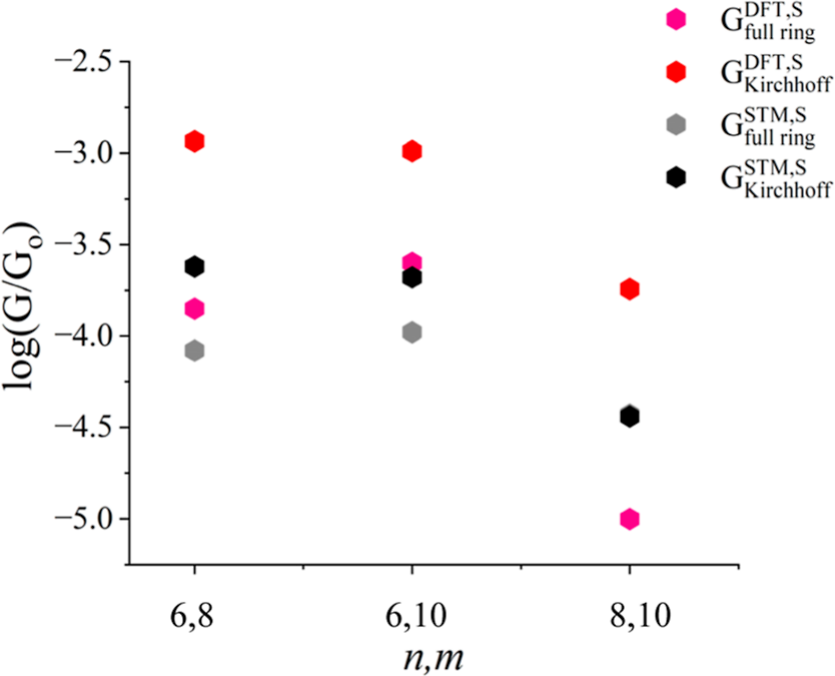
Validation of Kirchhoff’s law using asymmetric cycloalkanes of thiol anchors theoretically and experimentally. A logarithmic conductance of full rings was compared with a logarithmic conductance derived from Kirchhoff’s law. The red hexagons represent DFT Kirchhoff’s formula *G* values, while pink hexagons represent DFT *G* values for full rings with a thiol anchor. The black hexagons indicate STM Kirchhoff’s formula *G* values, while the gray hexagons indicate STM *G* values for full rings with the same anchor. Note: The experimental measurements are described in ref (27).

In Figure 2 above, we compared our DFT simulations against the STM measurements for symmetric cyclic molecules (i.e., the −*SH* end group). Similarly, Figure 4 compares our DFT simulations against STM measurements for asymmetric cyclic molecules with thiol anchor groups. As shown in Figure 4, DFT conductance values are more significant than STM conductance values when Kirchhoff’s formula is applied to asymmetric cyclic molecules containing thiol groups. Regarding full rings, the DFT calculations are slightly higher than those obtained from STM measurements. We observe excellent agreement between theory and experiment, demonstrating the validity of our DFT results. The logarithmic conductance values of full rings are lower than those determined by Kirchhoff’s law. Hence, Kirchhoff’s law cannot be applied to the nanoscale cycloalkane structures, either for symmetric or asymmetric rings.

## 4 Conclusions

The electron transport properties of symmetric and asymmetric alkane cyclic (*SAC* and *AAC*) molecules and their corresponding linear chains with three different terminal end groups including thiol (−*SH*), direct carbon (−*C*), and amine (−*NH*_2_) have been extensively studied, revealing limitations in applying Kirchhoff’s law due to their dimensions scale (i.e., nanoscale structures).

The strong dependence of Kirchhoff’s law on *SAC* and *AAC* size, corroborated by experimental measurements, underscores the influence of quantum interference in these molecular scale structures. This effect limited the validity of Kirchhoff’s law at tiny systems, enabling significant modulation of electron transport under a quantum interference effect.

Nevertheless, this proof-of-principle study opens new ideas for designing electronic and thermoelectric devices based on quantum interference of multibranch and conjugated molecules, with potential practical applications.

## Supporting Information Available

Theoretical simulations, including geometry of isolated cyclic alkane molecules; binding energy of four terminal groups on gold, optimized DFT structures of compounds in their junctions; conductance comparison between cyclic molecules of different terminal groups; and validation of Kirchhoff’s law in the nanoscale structures with cyclic molecules (PDF)
