# Supplementary material for: Does Kirchhoff’s Law Work in Molecular-Scale Structures?
Source: ACS Omega. 2025 Feb 27;10(9):9314–20. doi: 10.1021/acsomega.4c09854 (PMC11904646; doi:10.1021/acsomega.4c09854)
Supplement: Supplementary file 1 — ao4c09854_si_001.pdf [file ao4c09854_si_001.pdf]

Does Kirchhoff's law work in molecular-scale structures?

Abdullah Alshehab[a] and Ali K. Ismael [b*]

[a] Physics Department, College of Science, King Faisal University, Al Ahsa, Saudi Arabia

[b] Physics Department, Lancaster University, Lancaster, LA1 4YB, UK

1. Theoretical details

1.1 Geometry of isolated cyclic alkane molecules

As shown in Tables S1-S6, DFT code (SIESTA)[1-6] was used to obtain fully relaxed geometries for isolated cyclic molecules.Tables S1-S2 present 17 symmetric and asymmetric cyclic alkane molecules terminated with direct carbon in their fully relaxed and isolated conformations. Consider (odd-odd) rings as an example of symmetric molecules. We began with (C_3C_3) and then increased the length by adding two *CH2* units for each branch until n =9 (C_9C_9). This is also true for (even-even) rings. However, we began with (C_4C_4) and continued until ($C_{10}C_{10}$), as shown in Table S1. In the case of asymmetric cyclics, as shown in Table S2, such as (odd-odd+2) molecules, we start with (C_3C_5) and then add two *CH2* units for each branch until n=9, m=11 (C_9C_{11}). The same story applies to (even-even+2) molecules, starting with (C_4C_6) and ending with ($C_{10}C_{12}$).

The story is the same in Tables S3-S4; however, symmetric and asymmetric cyclic molecules are now terminated with thiol anchors, respectively.Tables S5-S6 show that cyclic molecules are now terminated with amine anchors.

Table S1. Completely relaxed cyclic molecules containing direct carbon. The (odd-odd) and (even-even) molecules represent symmetric cyclic molecules.

**Symmetric-Direct C anchor**			
**Molecule**	**Odd-odd**	**Molecule**	**Even-even**
Cyclooctane (ref.7) (C_3C_3)	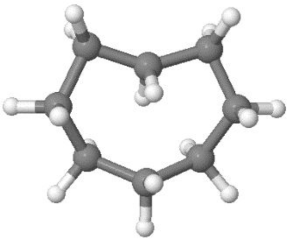	Cyclodecane (ref.10) (C_4C_4)	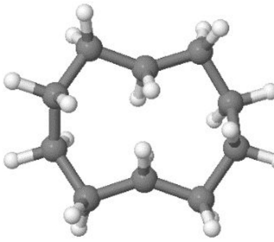
Cyclododecane (ref.8) (C_5C_5)	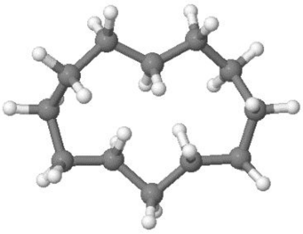	Cyclotetradecane (ref.11) (C_6C_6)	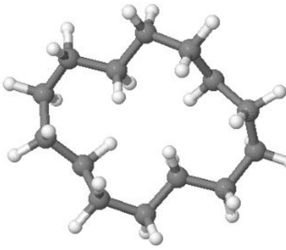
Cyclohexadeane (ref.9) (C_7C_7)	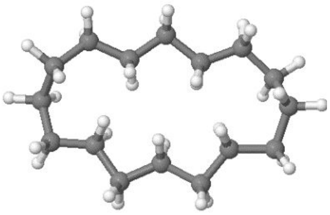	Cyclooctadecane (ref.9) (C_8C_8)	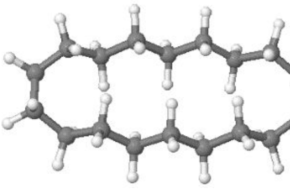
Cycloeicosane (ref.9) (C_9C_9)	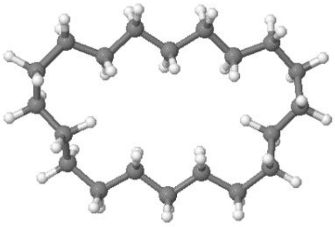	Cyclodocosane (ref.12) ($C_{10}C_{10}$)	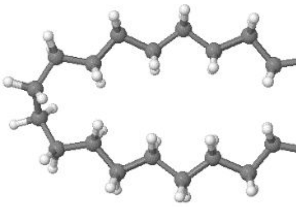

Table S2. Completely relaxed asymmetric cyclic molecules containing direct carbon. The (odd-odd+2) and (even-even+2) molecules refer to asymmetric cyclic molecules.

Asymmetric-Direct C anchor			
**Molecule**	**Odd-odd+2**	**Molecule**	**Even-even+2**
Cyclooctane (ref.10) (C_3C_5)	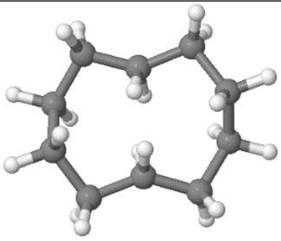	Cyclododecane (ref.8) (C_4C_6)	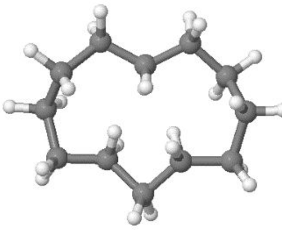
Cyclotetradecane (ref.11) (C5C7)	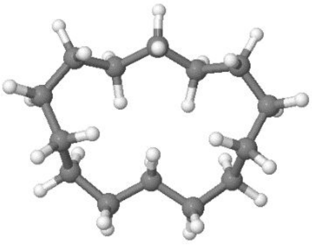	Cyclohexadecane (ref.9) (C_6C_8)	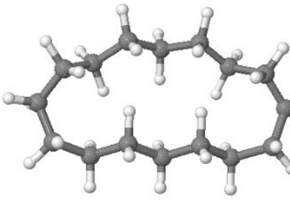
Cyclooctadecane (ref.9) (C_7C_9)	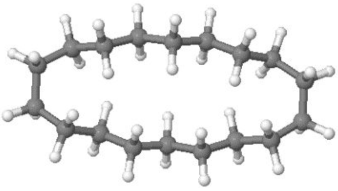	Cyclooctadecane (ref.9) (C_6C_{10})	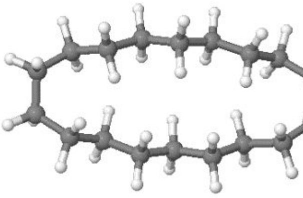
Cyclodocosane (ref.12) (C_9C_{11})	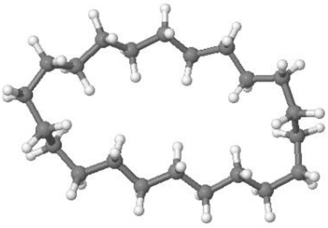	Cycloeicosane (ref.9) (C_8C_{10})	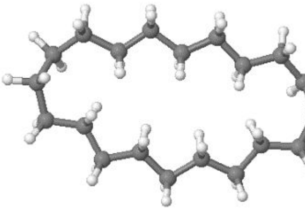
		Cyclotetracosane (ref.13) ($C_{10}C_{12}$)	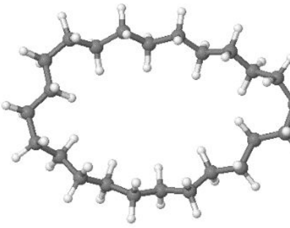

Table S3. Completely relaxed symmetric cyclic molecules with thiol anchor. The (odd-odd) and (even-even) molecules represent symmetric cyclic molecules.

Symmetric-Thiol anchor			
**Molecule**	**Odd-odd**	**Molecule**	**Even-eve[illegible]**
1,5-Dithiocane<br>(ref.14)<br>(C_3C_3)	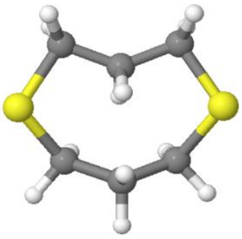	1,6-dithiecane<br>(ref.17)<br>(C_4C_4)	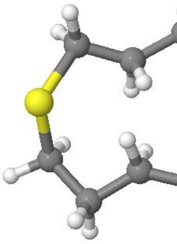
1,7-Dithiacyclododecane<br>(ref.15)<br>(C_5C_5)	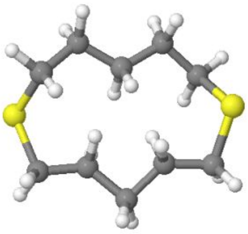	1,8-Dithia-cyclotetradecan<br>(ref.18-19)<br>(C_6C_6)	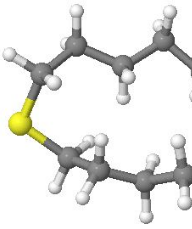
1,9-Dithia-cyclohexadecan<br>(ref.16)<br>(C_7C_7)	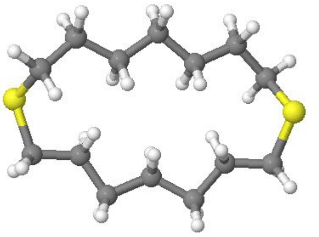	1,10-Dithia-cyclooctadecan<br>(ref.19)<br>(C_8C_8)	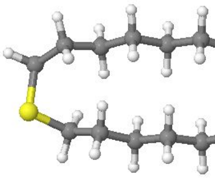
(C_9C_9)	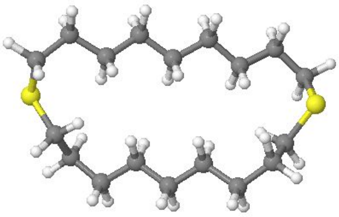	($C_{10}C_{10}$)<br>(ref.19)	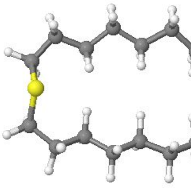

Table S4. Completely relaxed cyclic molecules with thiol anchor. The (odd-odd+2) and (even-even+2) rings refer to asymmetric cyclic molecules.

Asymmetric-Thiol anchor			
**Molecule**	**Odd-odd+2**	**Molecule**	**Even-even+2**
(C_3C_5)	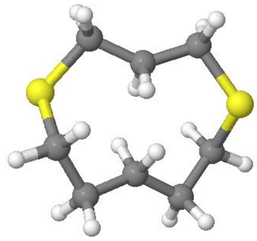	1,6-Dithiacyclododecane (ref.20) (C_4C_6)	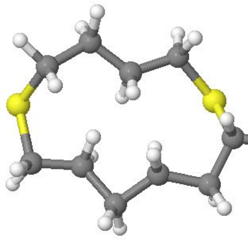
(C_5C_7)	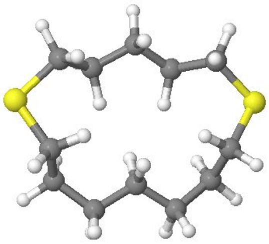	(C_6C_8) (ref.19)	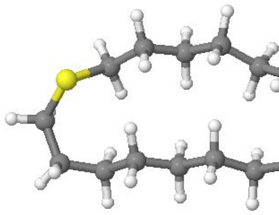
(C_7C_9)	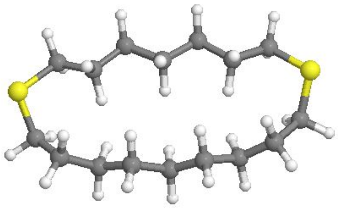	1,8-Dithiacyclooctadecane (ref.19) (C_6C_{10})	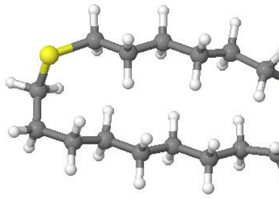
(C_9C_{11})	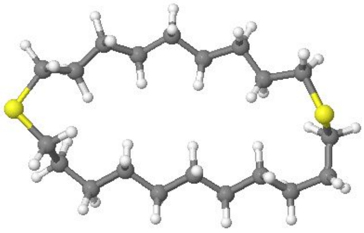	(C_8C_{10}) (ref.19)	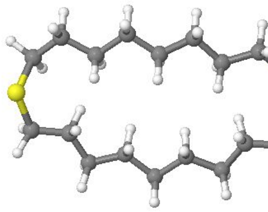
		($C_{10}C_{12}$)	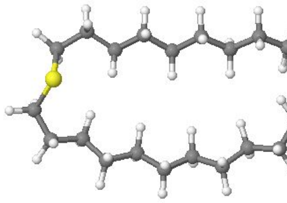

Table S5. Completely relaxed cyclic molecules with amine anchor. The (odd-odd) and (even-even) rings represent symmetric cyclic molecules.

Symmetric-Amine anchor			
**Molecule**	**Odd-odd**	**Molecule**	**Even-eve**
1,5-Diazacyclooctane (ref.21) (C_3C_3)	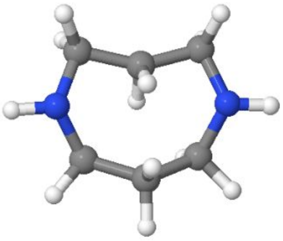	1,6-Diazecane (ref.25) (C_4C_4)	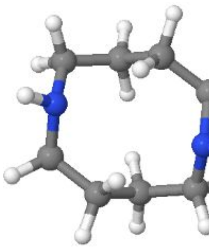
1,7-Diazacyclododecane (ref.22) (C_5C_5)	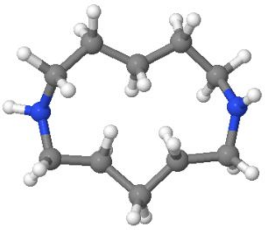	1,8-Diazacyclotetradecan (ref.26) (C_6C_6)	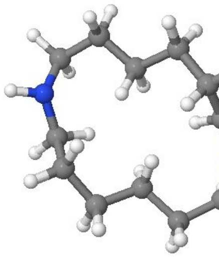
1,9-Diazacyclohexadecane (ref.23) (C_7C_7)	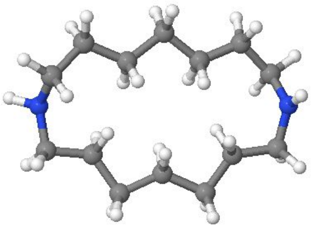	1,10-Diazacyclooctadecane (ref.27) (C_8C_8)	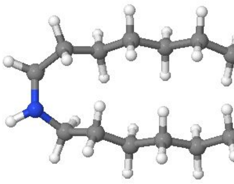
1,11-Diaza-cycloeikosan (ref.24) (C_9C_9)	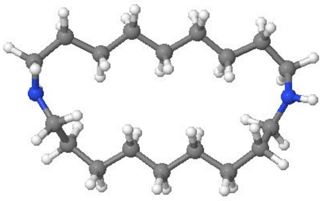	1,12-Diazacyclodocosane (ref.28) ($C_{10}C_{10}$)	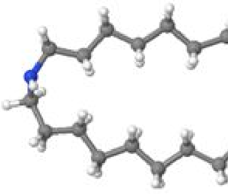

Table S6. Completely relaxed cyclic molecules with amine anchor. The (odd-odd+2) and (even-even+2) rings refer to asymmetric cyclic molecules.

Asymmetric-Amine anchor

Molecule	Odd-odd+2	Molecule	Even-even-
1,5-Diazecane (ref.29) (C_3C_5)	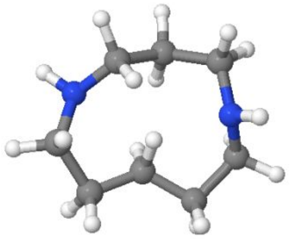	1,6-Diazacyclododecane (ref.31) (C_4C_6)	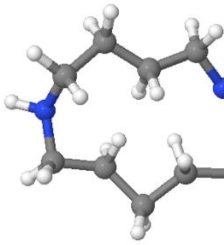
1,7-Diazacyclotetradecane (ref.29) (C_5C_7)	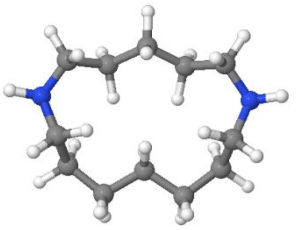	(C_6C_8)	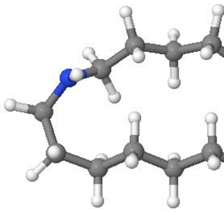
(C_7C_9)	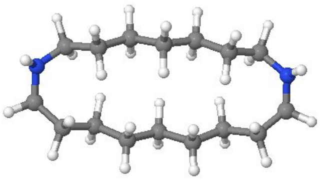	1,8-Diazacyclooctadecane (ref.32) (C_6C_{10})	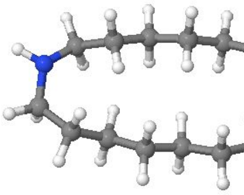
(C_9C_{11})	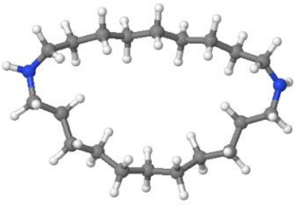	(C_8C_{10})	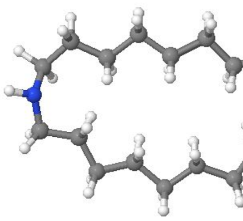
		($C_{10}C_{12}$)	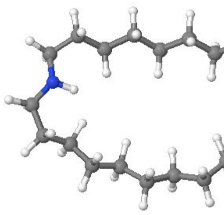

2. Binding energy of four terminal groups on gold

To determine the optimal distance between the cyclic molecules bound to the gold surfaces with three different terminal groups (Au-NH_2, Au-S, and Au-C), DFT and the counterpoise method were used, thereby eliminating basis set superposition errors (BSSE). The binding distance is determined by measuring the distance between the gold surface and the molecule's terminated end group or atom. Using SIESTA, the ground state energy of the overall system was calculated and is denoted E_{AB}^{AB}. The gold leads are composed of six layers containing 30 atoms each. Using ghost atoms in

SIESTA, the energy of each monomer was then calculated on a fixed basis. So, in the presence of the fixed basis, the energy of each molecule is defined as E_A^{AB}, and for the isolated gold, it is defined as E_B^{AB}. As a result, the binding energy can be calculated as follows: [1,5]

$$\text{Binding Energy} = E_{AB}^{AB} - E_A^{AB} - E_B^{AB} \quad \text{(S1)}$$

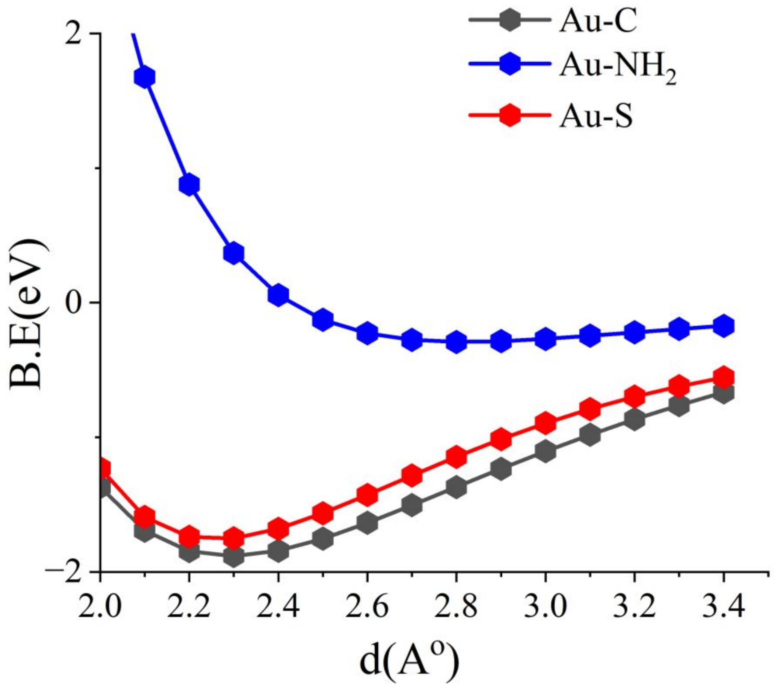

Figure S1. Binding energy of cyclic molecules to gold as a function of molecule-contact distance. The equilibrium distance (i.e. the minimum of the binding energy curve) is found to be approximately 2.3, 2.3 and 2.8 Å for Au-C, Au-S,and Au- NH_2 (top to bottom).

3. Optimised DFT structures of compounds in their Junctions

Here are a few examples of optimised DFT structures of the cyclic molecules, in their junctions as shown in Figure S2.

a

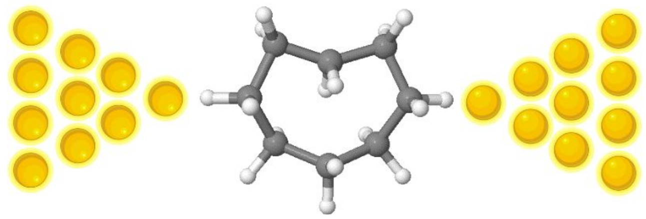

b

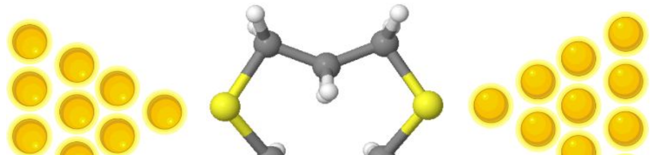

Figure S2. Examples of cyclic molecules in Au|cyclic molecules|Au junctions: **(a), (b)** and **(c):** A double-branch n = 3 cyclic molecules with (Au-C), (Au-S) and (Au-NH_2) repectively.

4. Conductance comparison between cyclic molecules of different terminal groups

In this section, we compare the conductance G of cyclic molecules of three different terminal groups, including direct carbon Au-C, thiols Au-S and amine Au-NH_2. Figures S3-S6 present transmission coefficient curves for cyclic molecules with Au-C, whereas Figures S7-S10 show the same transmission curves but with Au-S. A final set of

transmission coefficient curves with terminal groups Au-NH_2 is presented in Figures S11-S14.

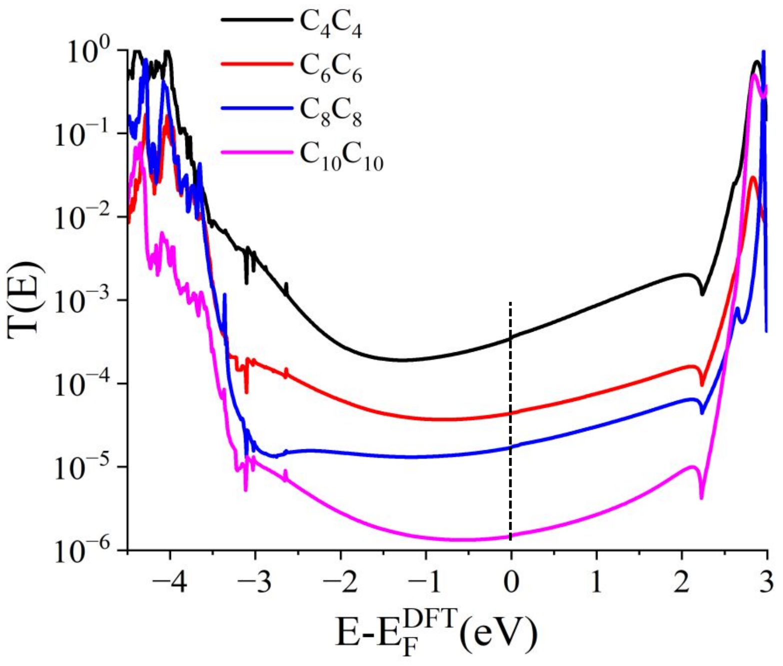

Figure S3. Transmission coefficient curves of cyclic molecules with (Au-C). Even-even cyclic molecules transmission coefficients T(E) against electron energy E for C_4C_4 (black line), C_6C_6 (red line), C_8C_8 (blue line) and $C_{10}C_{10}$ (pink line).

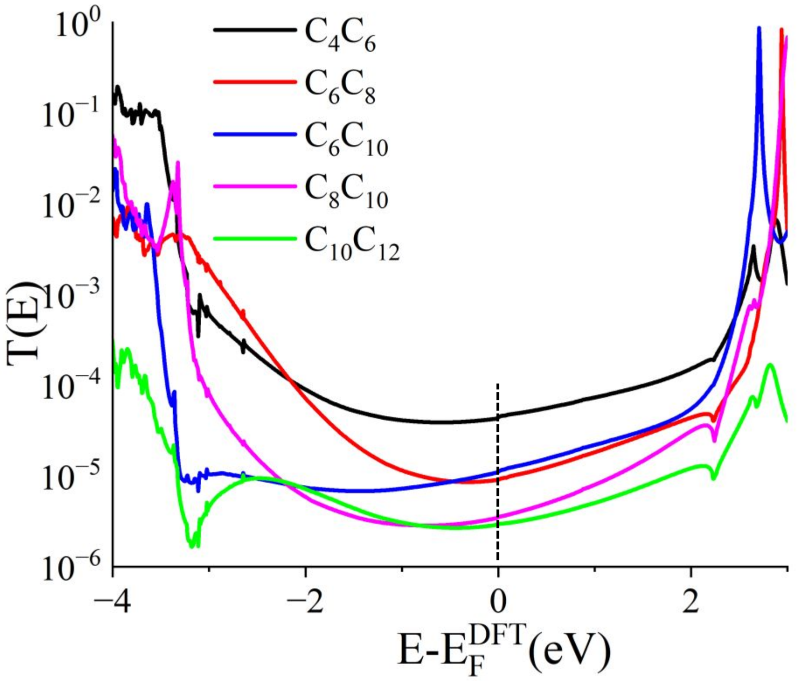

Figure S4. Transmission coefficient curves of cyclic molecules molecules with (Au-C)**.** Even-even+2 cyclic molecules molecules transmission coefficients T(E) against electron energy E for C_4C_6 (black line), C_6C_8 (red line), C_6C_{10} (blue line), C_8C_{10} (pink line) and $C_{10}C_{12}$ (green line).

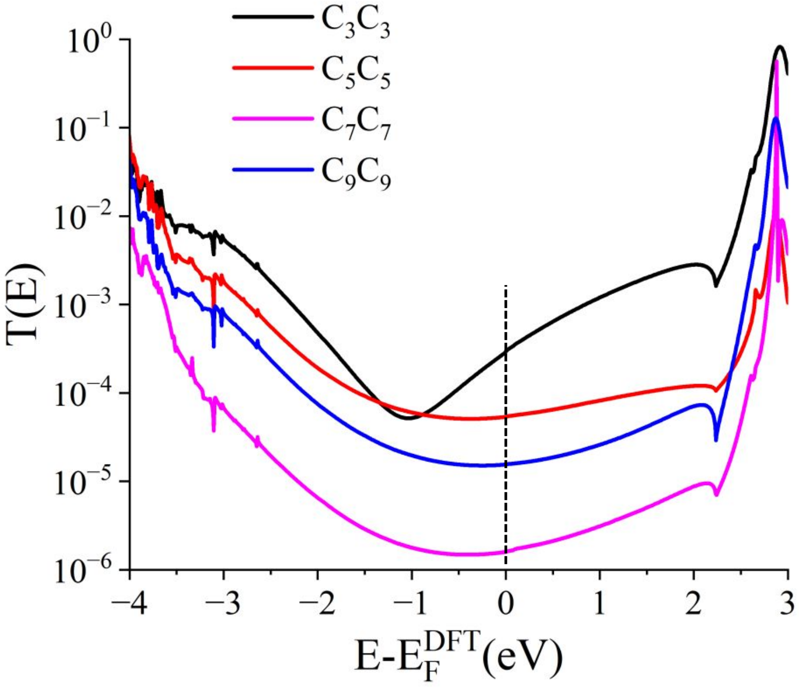

Figure S5. Transmission coefficient curves of cyclic molecules with (Au-C). Odd-odd cyclic molecules transmission coefficients T(E) against electron energy E for C_3C_3 (black line), C_5C_5 (red line), C_7C_7 (pink line) and C_9C_9 (blue line).

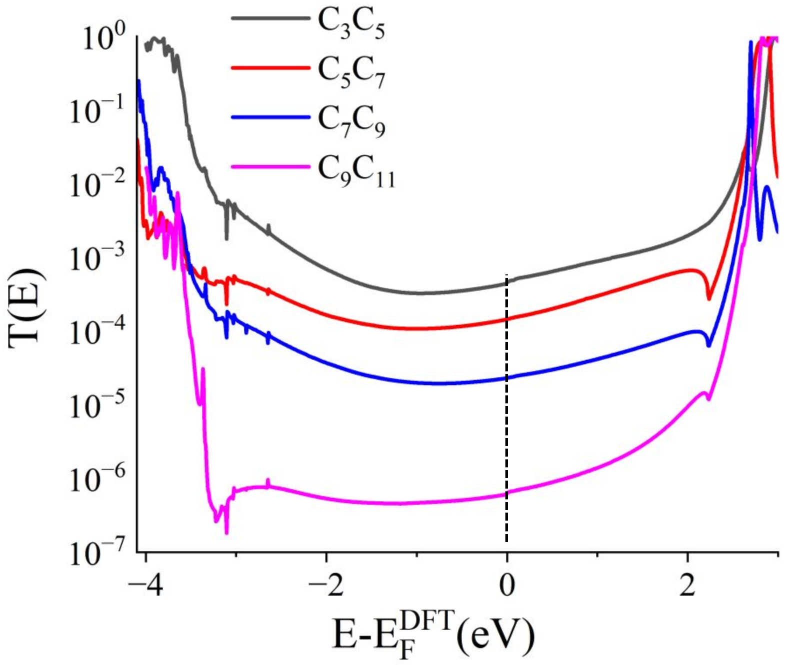

Figure S6. Transmission coefficient curves of cyclic molecules with (Au-C). Odd-odd+2 cyclic molecules transmission coefficients T(E) against electron energy E for C_3C_5 (black line), C_5C_7 (red line), C_7C_9 (blue line) and C_9C_{11} (pink line).

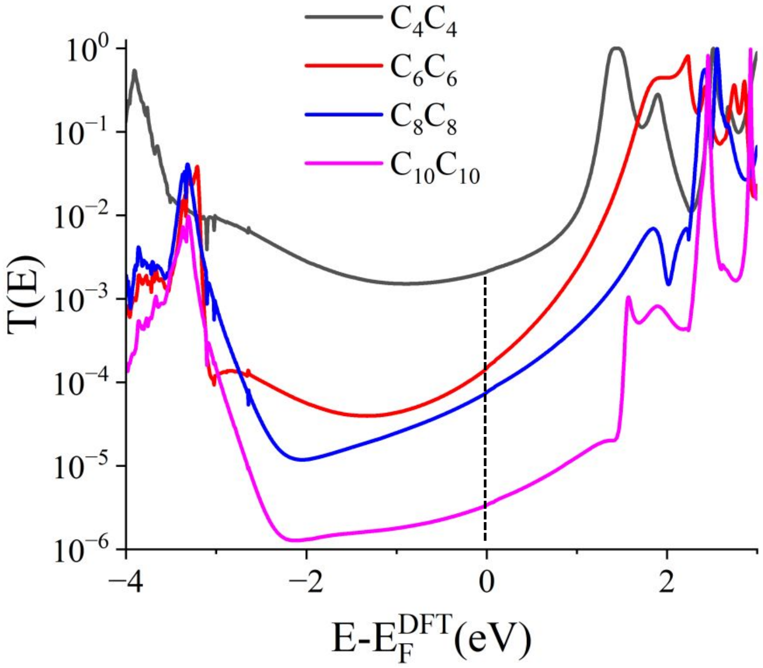

Figure S7. Transmission coefficient curves of cyclic moleculeswith (Au-S)**.** Even-even cyclic molecules transmission coefficients T(E) against electron energy E for C_4C_4 (black line), C_6C_6 (red line), C_8C_8 (blue line) and $C_{10}C_{10}$ (pink line).

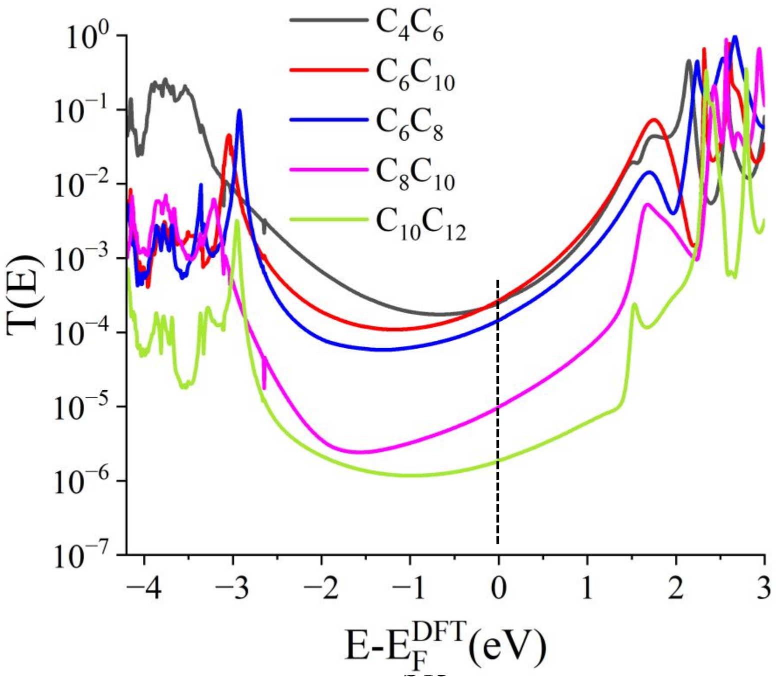

Figure S8. Transmission coefficient curves of cyclic molecules with (Au-S). Even-even+2 cyclic molecules transmission coefficients T(E) against electron energy E for C_4C_6 (black line), C_6C_{10} (red line), C_6C_8 (blue line), C_8C_{10} (pink line) and $C_{10}C_{12}$ (green line).

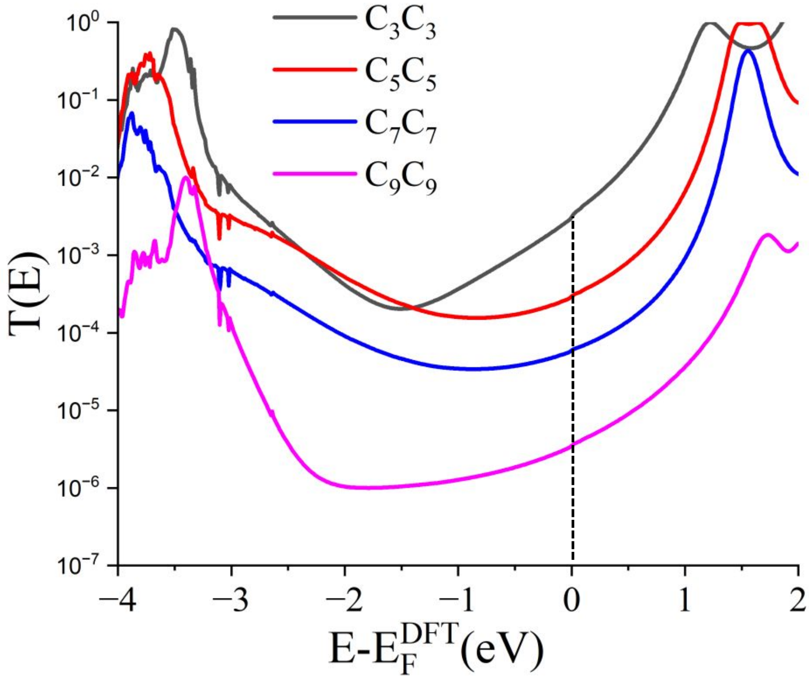

Figure S9. Transmission coefficient curves of cyclic molecules with (Au-S). Odd-odd cyclic molecules transmission coefficients T(E) against electron energy E for C_3C_3 (black line), C_5C_5 (red line), C_7C_7 (blue line) and C_9C_9 (pink line).

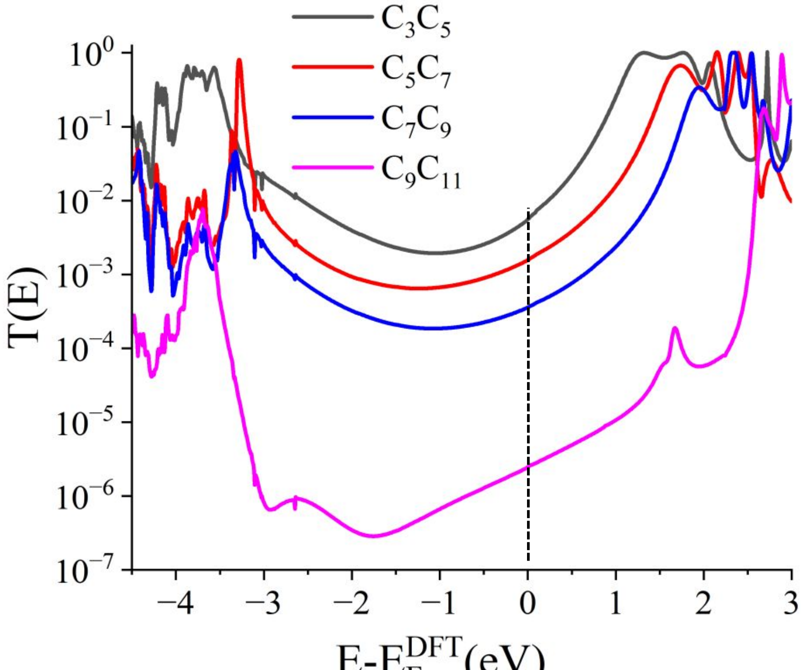

Figure S10. Transmission coefficient curves of cyclic molecules with (Au-S). Odd-odd+2 cyclic molecules transmission coefficients T(E) against electron energy E for C_3C_5 (black line), C_5C_7 (red line), C_7C_9 (blue line) and C_9C_{11} (pink line).

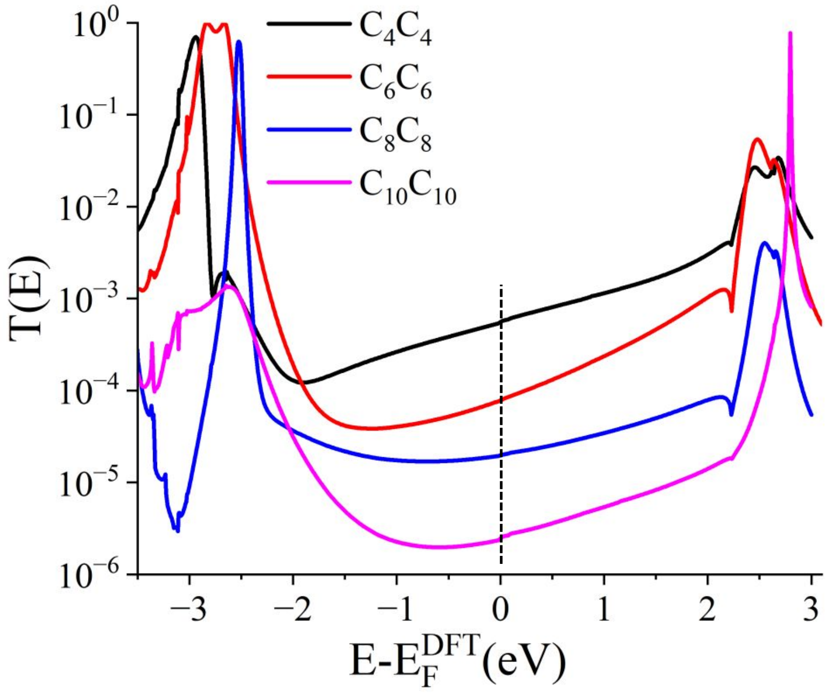

Figure S11. Transmission coefficient curves of cyclic molecules with (Au-NH_2). Even-even cyclic molecules transmission coefficients T(E) against electron energy E for C_4C_4 (black line), C_6C_6 (red line), C_8C_8 (blue line) and $C_{10}C_{10}$ (pinkline).

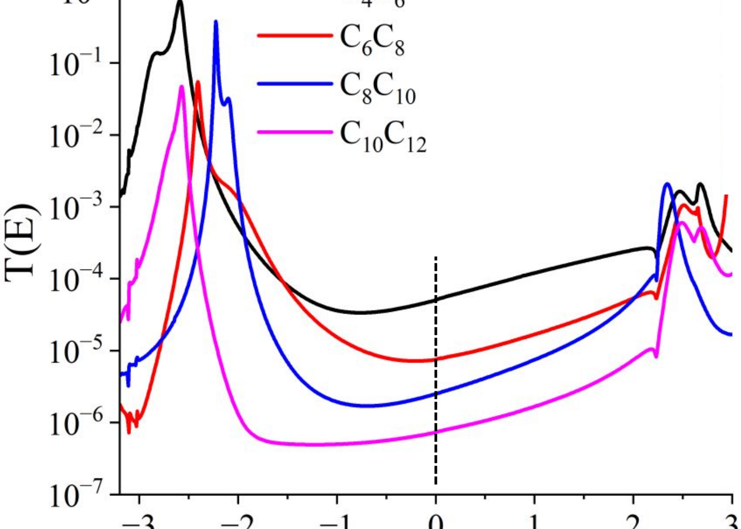

Figure S12. Transmission coefficient curves of cyclic molecules with (Au-NH_2)**.** Even-even+2 cyclic molecules transmission coefficients T(E) against electron energy E for C_4C_6 (black line), C_6C_{10} (red line), C_6C_8 (blue line), C_8C_{10} (pink line) and $C_{10}C_{12}$ (green line).

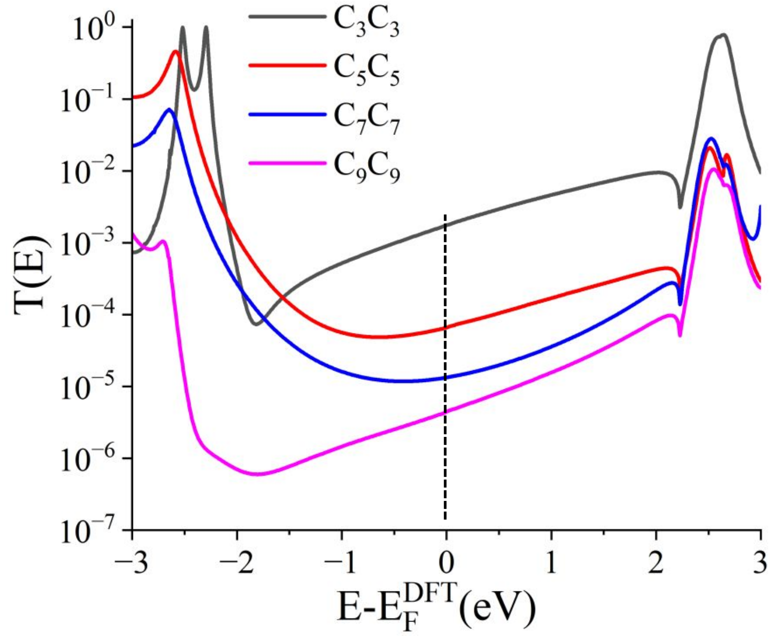

Figure S13. Transmission coefficient curves of cyclic molecules with (Au-NH_2)**.** Odd-odd cyclic molecules transmission coefficients T(E) against electron energy E for C_3C_3 (black line), C_5C_5 (red line), C_7C_7 (blue line) and C_9C_9 (pink line).

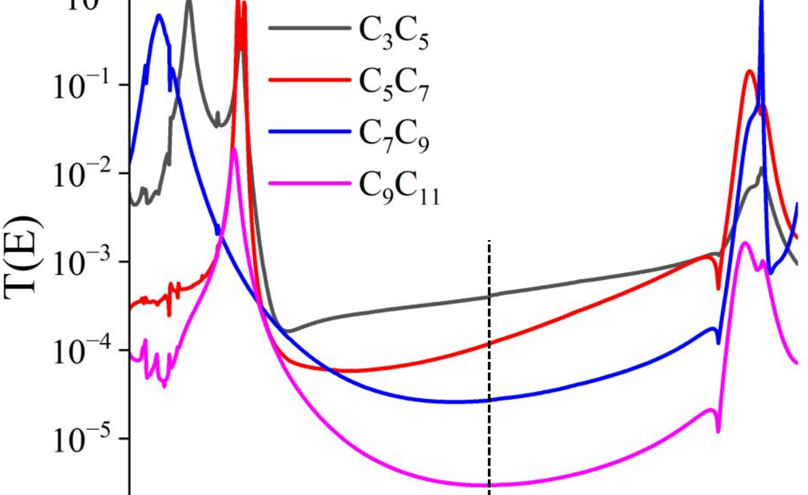

Figure S14. Transmission coefficient curves of cyclic molecules with (Au-NH_2)**.** Odd-odd+2 cyclic molecules transmission coefficients T(E) against electron energy E for C_3C_5 (black line), C_5C_7 (red line), C_7C_9 (blue line) and C_9C_{11} (pink line).

5. Validation of Kirchhoff's law in the nanoscale structures with cyclic molecules

Here is further evidence indicating that Kirchhoff's law fails to hold at the nanoscale with sigma systems. According to Table S7, conductance calculations are shown with amine anchors, whereas Table S8 shows the same story with a direct carbon anchor. Again, we have additional evidence that Kirchhoff's formula is invalid in the nanoscale world. The total conductance of each individual branch when Kirchhoff's law is applied (fourth column in Tables S7-S8) is greater than the predicted DFT values (last column with the same Tables).

Table S7. Conductances of linear alkane chains and symmetric cyclic molecules were determined from DFT calculations and experimental measurements (STM) with an amine anchor. The third and fifth columns represent the DFT calculations from ref.33, while the fourth and sixth columns represent experimental results from ref.34.

Anchor	*n*	DFT $\log(G/G_\circ)$	STM $\log(G/G_\circ)$	DFT $G = G_1 + G_2$	STM $G = G_1 + G_2$	*n,m*	DFT $\log(G/G_\circ)$
	3	-2.72	=	-2.40	=	3,3	-2.76
	4	-3.00	-3.04	-2.69	-2.69	4,4	-3.22
	5	-3.50	=	-3.19	=	5,5	-4.18
-NH_2	6	-4.00	-4.00	-3.70	-3.52	6,6	-4.10
	7	-4.30	=	-4.00	=	7,7	-4.87
	8	-4.90	-5.00	-4.60	-4.00	8,8	-4.70
	9	-5.31	=	-5.00	=	9,9	-5.34
	10	-5.70	-6.00	-5.40	-5.00	10,10	-5.60

Table S8. Conductances of linear alkane chains and symmetric cyclic molecules were determined from DFT calculations and experimental measurements (STM) with a direct carbon anchor. The third and fifth columns represent the DFT calculations from ref.33, while the fourth and sixth columns represent experimental results from refs.35-38.

Anchor	n	DFT $\log(G/G_\circ)$	STM $\log(G/G_\circ)$	DFT $G = G_1 + G_2$	STM $G = G_1 + G_2$	n,m	DFT $\log(G/G_\circ)$
	3	-0.70	=	-0.40	=	3,3	-3.50
	4	-0.92	-2.00	-0.62	-1.69	4,4	-3.50
	5	-1.70	=	-1.40	=	5,5	-4.30
-C	6	-2.00	-2.69	-1.69	-2.39	6,6	-4.40
	7	-2.50	=	-2.19	=	7,7	-4.80
	8	-3.00	-3.50	-2.69	-3.19	8,8	-4.80
	9	-3.40	=	-3.10	=	9,9	-5.70
	10	-4.00	=	-3.69	=	10,10	-5.80

As shown in Tables S7-S8, Kirchhoff's equation and our DFT calculations are related mathematically as follows:

$$G_{ring}^{P,NH_2} \approx 0.88\, G_{kirchhoff}^{DFT,NH_2} - 0.98 \qquad \text{(S2)}$$

With amine terminal groups, G_{ring}^{P,NH_2} and $G_{kirchhoff}^{DFT,NH_2}$ represent the predicted conductance values and Kirchhoff's law resulted conductance values, respectively

$$G_{ring}^{P,C} \approx 0.73\, G_{kirchhoff}^{DFT,C} - 3.1621 \qquad \text{(S3)}$$

Again, $G_{ring}^{P,C}$represents the predicted conductance values and $G_{ring}^{DFT,C}$ represents Kirchhoff's law resulted conductance values for amine terminal groups.

References

1. Kobko, N., & Dannenberg, J. J. (2001). Effect of Basis Set Superposition Error (BSSE) upon ab Initio Calculations of Organic Transition States. *The Journal of Physical Chemistry A*, *105*(10), 1944–1950.

2. Kohn, W., & Sham, L. J. (1965). Self-Consistent Equations Including Exchange and Correlation Effects. *Physical Review*, *140*(4A), A1133–A1138.

3. Perdew, J. P., Burke, K., & Ernzerhof, M. (1996). Generalized Gradient Approximation Made Simple. *Physical Review Letters*, *77*(18), 3865–3868.

4. Perdew, J. P., & Zunger, A. (1981). Self-interaction correction to density-functional approximations for many-electron systems. *Physical Review. B, Condensed Matter*, *23*(10), 5048–5079.

5. Sinnokrot, M. O., Valeev, E. F., & Sherrill, C. D. (2002). Estimates of the Ab Initio Limit for π−π Interactions: The Benzene Dimer. *Journal of the American Chemical Society*, *124*(36), 10887–10893.

6. Soler, J. M., Artacho, E., Gale, J. D., García, A., Junquera, J., Ordejón, P., & Sánchez-Portal, D. (2002). The SIESTA method forab initioorder-Nmaterials simulation. *Journal of Physics Condensed Matter*, *14*(11), 2745–2779.

7. Cao, Y., Huang, C., & Lu, Q. (2024). Photoelectrochemically driven iron-catalysed C(sp3)−H borylation of alkanes. *Nature Synthesis*, *3*(4), 537–544.

8. Ding, W. W., He, Z. Y., Sayed, M., Zhou, Y., Han, Z. Y., & Gong, L. Z. (2024). Enantioselective synthesis of β- and α-amino ketones through reversible alkane carbonylation. *Nature Synthesis*, *3*(4), 507–516.

9. Dragojlovic, V. Conformational analysis of cyclics. *ChemTexts* **1**, 14 (2015).

10. Ahluwalia, V., & Aggarwal, R. (2023). *Alicyclic Chemistry*. Springer Nature.

11. Chickos, J. S., Hesse, D. G., Panshin, S. Y., Rogers, D. W., Saunders, M., Uffer, P. M., & Liebman, J. F. (1992). The strain energy of cyclotetradecane is small. *Journal of Organic Chemistry*, *57*(6), 1897–1899.

12. Meng, X., Lu, H., Zhang, Z., Peng, P., & Volkman, J. K. (2023). Structural characterization and mass spectrometry fragmentation signatures of macrocyclic alkanes isolated from a Sydney Basin torbanite, Australia. *Acta Geochimica,* 42(3), 488–494.

13. Swain, S., Bej, S., Bishoyi, A.K. *et al.* Recent progression on phytochemicals and pharmacological properties of the filamentous cyanobacterium *Lyngbya* sp.. *Naunyn-Schmiedeberg's Arch Pharmacol* **396**, 2197–2216 (2023).

14. Zhang, S., Wang, X., Su, Y., Qiu, Y., Zhang, Z., & Wang, X. (2014). Isolation and reversible dimerization of a selenium–selenium three-electron σ-bond. *Nature Communications*, *5*(1).

15. National Center for Biotechnology Information (2024). PubChem Compound Summary for CID 12560936, 1,7-Dithiacyclododecane. Retrieved July 9, 2024 from https://pubchem.ncbi.nlm.nih.gov/compound/1_7-Dithiacyclododecane.

16. Müller, A., Funder-Fritzsche, E., Konar, W. *et al.* Thia- und Dithia-cyclic und die Spaltung einiger gesättigter Heterocyclen mit Methyljodid. *Monatshefte für Chemie* **84**, 1206–1220 (1953).

17. Glass, R. S. (1990). Design, Synthesis, and Conformational Analysis of Compounds Tailored for the Study of Sulfur-Centered Reactive Intermediates. In *Springer eBooks* (pp. 227–238).

18. Müller, A., Funder-Fritzsche, E., Konar, W. *et al.* Thia- und Dithia-cyclic und die Spaltung einiger gesättigter Heterocyclen mit Methyljodid. *Monatshefte für Chemie* **84**, 1206–1220 (1953).

19. Ye, J., Al-Jobory, A., Zhang, Q. C., Cao, W., Alshehab, A., Qu, K., Alotaibi, T., Chen, H., Liu, J., Ismael, A. K., Chen, Z. N., Lambert, C. J., & Hong, W. (2022). Highly insulating alkane rings with destructive σ-interference. *Science China Chemistry*, *65*(9), 1822–1828.

20. National Center for Biotechnology Information (2024). PubChem Compound Summary for CID 544123, 1,6-Dithiacyclododecane. Retrieved July 9, 2024 from https://pubchem.ncbi.nlm.nih.gov/compound/1_6-Dithiacyclododecane.

21. Cheng, Y., & Qin, D. (2024). *Classification of Diverse Novel Alkaloids* (pp. 117–149).

22. National Center for Biotechnology Information (2024). PubChem Compound Summary for CID 14290132, 1,7-Diazacyclododecane. Retrieved September 2, 2024 from https://pubchem.ncbi.nlm.nih.gov/compound/1_7-Diazacyclododecane.

23. National Center for Biotechnology Information (2024). PubChem Compound Summary for CID 19890075, 1,9-Diazacyclohexadecane. Retrieved September 2, 2024 from https://pubchem.ncbi.nlm.nih.gov/compound/1_9-Diazacyclohexadecane.

24. Müller, A., Šrepel, E., Funder-Fritzsche, E. *et al.* Aza- und Diaza-cyclic. *Monatshefte für Chemie* **83**, 386–393 (1952).

25. Alder, R. W. (2005). Design of C2-Chiral Diamines That Are Computationally Predicted To Be a Million-fold More Basic than the Original Proton Sponges. *Journal of the American Chemical Society*, *127*(21), 7924–7931.

26. Mikhura, I.V., Formanovskii, A.A. Synthesis of aza-crown compounds by intramolecular cyclization of ω-amino acids. *Chem Heterocycl Compd* **28**, 205–212 (1992).

27. Müller, A., Funder-Fritzsche, E., Konar, W. *et al.* Thia- und Dithia-cyclic und die Spaltung einiger gesättigter Heterocyclen mit Methyljodid. *Monatshefte für Chemie* **84**, 1206–1220 (1953).

28. National Center for Biotechnology Information (2024). PubChem Compound Summary for CID 154128457, 1,12-Diazacyclodocosane. Retrieved September 2, 2024 from https://pubchem.ncbi.nlm.nih.gov/compound/1_12-Diazacyclodocosane.

29. National Center for Biotechnology Information (2024). PubChem Compound Summary for CID 12793068, 1,5-Diazecane. Retrieved September 3, 2024 from https://pubchem.ncbi.nlm.nih.gov/compound/1_5-Diazecane.

30. National Center for Biotechnology Information (2024). PubChem Compound Summary for CID 70649771, 1,7-Diazacyclotetradecane. Retrieved September 3, 2024 from https://pubchem.ncbi.nlm.nih.gov/compound/1_7-Diazacyclotetradecane.

31. National Center for Biotechnology Information (2024). PubChem Compound Summary for CID 122550382, 1,6-Diazacyclododecane. Retrieved September 3, 2024 from https://pubchem.ncbi.nlm.nih.gov/compound/1_6-Diazacyclododecane.

32. National Center for Biotechnology Information (2024). PubChem Compound Summary for CID 13128616, 1,8-Diazacyclooctadecane. Retrieved September 3, 2024 from https://pubchem.ncbi.nlm.nih.gov/compound/1_8-Diazacyclooctadecane.

33. Alshehab, A., & Ismael, A. K. (2023). Impact of the terminal end-group on the electrical conductance in alkane linear chains. *RSC Advances*, *13*(9), 5869–5873.

34. Chen, F., Li, X., Hihath, J., Huang, Z., & Tao, N. (2006). Effect of Anchoring Groups on Single-Molecule Conductance: Comparative Study of Thiol-, Amine-, and Carboxylic-Acid-Terminated Molecules. *Journal of the American Chemical Society*, *128*(49), 15874–15881.

35. Widawsky, J. R., Chen, W., Vázquez, H., Kim, T., Breslow, R., Hybertsen, M. S., & Venkataraman, L. (2013). Length-Dependent Thermopower of Highly Conducting Au–C Bonded Single Molecule Junctions. *Nano Letters*, *13*(6), 2889–2894.

36. Introduction to Linear Regression Analysis. (2010). In *Monographs on statistics and applied probability* (pp. 1–16).
37. Chen, F., Li, X., Hihath, J., Huang, Z. & Tao, N. Effect of anchoring groups on single-molecule conductance: Comparative study of thiol-, amine-, and carboxylic-acid-terminated molecules. J Am Chem Soc 128, 15874–15881 (2006).
38. Widawsky, J. R. et al. Length-dependent thermopower of highly conducting Au-C bonded single molecule junctions. Nano Lett 13, 2889–2894 (2013).
